# PEEP-ZEEP technique: cardiorespiratory repercussions in mechanically ventilated patients submitted to a coronary artery bypass graft surgery

**DOI:** 10.1186/1749-8090-6-108

**Published:** 2011-09-13

**Authors:** Marcus Vinicius Herbst-Rodrigues, Vitor Oliveira Carvalho, José Otávio Costa Auler, Maria Ignez Zanetti Feltrim

**Affiliations:** 1 Physiotherapy Division, Heart Institute (InCor), University of Sao Paulo Medical School, Sao Paulo, Brazil; 2 Laboratório de Insuficiência Cardíaca e Transplante, Heart Institute (InCor), University of Sao Paulo Medical School, Sao Paulo, Brazil; 3 Anesthesiology Division, Heart Institute (InCor), University of Sao Paulo Medical School, Sao Paulo, Brazil

**Keywords:** physical therapy modalities, respiratory mechanics, artificial respiration, pulmonary gas exchange, cardiovascular surgical procedures

## Abstract

**Background:**

The PEEP-ZEEP technique is previously described as a lung inflation through a positive pressure enhancement at the end of expiration (PEEP), followed by rapid lung deflation with an abrupt reduction in the PEEP to 0 cmH_2_O (ZEEP), associated to a manual bilateral thoracic compression.

**Aim:**

To analyze PEEP-ZEEP technique's repercussions on the cardio-respiratory system in immediate postoperative artery graft bypass patients.

**Methods:**

15 patients submitted to a coronary artery bypass graft surgery (CABG) were enrolled prospectively, before, 10 minutes and 30 minutes after the technique. Patients were curarized, intubated, and mechanically ventilated. To perform PEEP-ZEEP technique, saline solution was instilled into their orotracheal tube than the patient was reconnected to the ventilator. Afterwards, the PEEP was increased to 15 cmH_2_O throughout 5 ventilatory cycles and than the PEEP was rapidly reduced to 0 cmH_2_O along with manual bilateral thoracic compression. At the end of the procedure, tracheal suction was accomplished.

**Results:**

The inspiratory peak and plateau pressures increased during the procedure (p < 0.001) compared with other pressures during the assessment periods; however, they were within lung safe limits. The expiratory flow before the procedure were 33 ± 7.87 L/min, increasing significantly during the procedure to 60 ± 6.54 L/min (p < 0.001), diminishing to 35 ± 8.17 L/min at 10 minutes and to 36 ± 8.48 L/min at 30 minutes. Hemodynamic and oxygenation variables were not altered.

**Conclusion:**

The PEEP-ZEEP technique seems to be safe, without alterations on hemodynamic variables, produces elevated expiratory flow and seems to be an alternative technique for the removal of bronchial secretions in patients submitted to a CABG.

## Background

To carry out cardiovascular surgeries, patients must be anesthetized, curarized, intubated, and be put under mechanical ventilatory assistance. After surgery, patients are taken to the intensive care unit (ICU) for monitoring hemodynamic instability, arrhythmias and bleeding, among other complications of the surgical procedure [1].

The most common cardiovascular postoperative complications are related to extracorporeal circulation (ECC) and its inflammatory reaction. It is well known that ECC affects the lungs causing alveolar edema and atelectasis, which can lead the patient to a longer period of mechanical ventilation [2]. Longer periods of endotracheal intubation impair mucociliary transport and make necessary the use of airway clearance techniques [3].

Tracheal suctioning is one of the most used methods for removing secretions from airways and its classical procedure consists of disconnection from mechanical ventilation, followed by insertion of a suction catheter into the trachea for tracheal suctioning under negative pressure [4]. Nevertheless, in some cases, tracheal suction is insufficient for a complete secretion removal due to an increased secretion volume and/or viscosity. In this case, extra techniques for removing secretions, in special the ones that promote an expiratory airflow increase are indicated [5].

Based on the idea of increasing expiratory airflow to remove secretion, a technique named PEEP-ZEEP has been previously proposed [6]. This technique consists in imposing a gradual positive end-expiratory pressure on the respiratory system (PEEP) until 15 cmH_2_O followed by an abrupt PEEP reduction to 0 cmH_2_O (ZEEP), in association with a manual bilateral thoracic compression to potentiate the increase of expiratory airflow. Despite this, the cardiorespiratory repercussions have never been studied.

The aim of this study was to analyze the repercussion of PEEP-ZEEP technique on the cardiorespiratory system and evaluate its safety in patients submitted to a coronary artery bypass graft surgery (CABG).

## Method

### Study population

Fifteen patients (11 men, 60 ± 8 years) in their first cardiac surgery were prospectively included in this study performed in a tertiary cardiology hospital (Heart Institute (InCor), University of Sao Paulo-Medical School, Sao Paulo, Brazil). Patients did not have pulmonary disease and all subjects underwent a CABG with extracorporeal circulation (Table 1). Patients who needed extracorporeal circulation more than 120 minutes, who showed hemodynamic instability (mean arterial pressure (MAP < 60 mmHg)) or the needed intra-aortic balloon assistance were excluded from this study. Were also excluded patients who showed S_pO2 _lower than 92% during the initial assessment.

**Table 1:** Patient's characteristics.

Patients - PEEP ZEEP technique	
Age (years)	60 ± 9
Weight (kg)	73 ± 13
Height (cm)	166 ± 9
Body mass index (Kg/m^2^)	27 ± 4
ECC (minutes)	90 ± 23
Homoglobin (g/dL)	10.66 ± 1.34
Hematocrit (%)	32.21 ± 4.1

ECC: Extracorporeal circulation. Values are presented as mean and standard deviation.

### Study design and cardiorespiratory measurements

**Figure 1:**
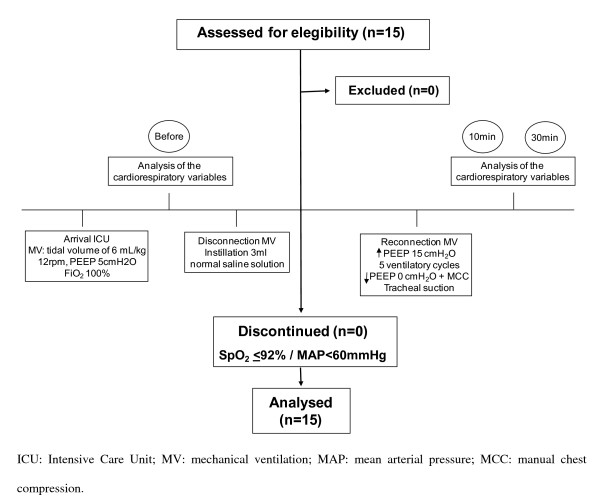
**Patient's flow and study design**.

This study began 30 minutes after patient's arrival at the postoperative intensive care unit. The patients were evaluated at 3 different moments: before the PEEP-ZEEP technique, 10 minutes and 30 minutes after the PEEP-ZEEP technique (Figure 1).

Heart rate (HR) and MAP (66-Hewlett Packard™ and Biomonitor7-BESE™) were used to evaluate the hemodynamic status. Patient's oxygenation was evaluated by peripheral saturation (S_pO2_). End-tidal carbon dioxide (ETCO_2_) and respiratory mechanics (peak inflation pressure (PIP), plateau pressure (Pplateau), inspiratory flow (Vinsp), expiratory flow (Vexp), inspiratory resistance (Rawinsp), expiratory resistance (Rawexp) and total static lung compliance (Cst)) were measured by a respiratory monitor CO_2_SMO™ DX8100™-Dixtal™.

Before PEEP-ZEEP technique, patients were in a supine position, sedated with propophol (1 to 3 mg/kg/weight) and curarized with atracurium bezylate (0.3 to 0.5 mg/kg/weight). Patients were in use of a mean arterial pressure catheter and were intubated and mechanically ventilated with Veolar™ or Amadeus™ (Hamilton Medical™, Switzerland) in an assisted/controlled (A/C) mode, with a tidal volume of 6 mL/kg/weight, respiratory rate of 12 bpm, PEEP of 5 cmH_2_O and fraction of inspired oxygen of 1.0.

### PEEP-ZEEP technique

Initially, the S_p_O_2 _was checked and then, patients were disconnected from the mechanical ventilator for the instillation of 3 mL of normal saline solution through the orotracheal tube, followed by reconnection to the mechanical ventilator. In sequence, PEEP was elevated to 15 cmH_2_O and the peak inspiratory pressure was limited to 40 cmH_2_O for security reasons throughout 5 respiratory cycles. After the fifth respiratory cycle, the PEEP was abruptly reduced to 0 cmH_2_O (ZEEP) in association with manual bilateral thoracic compression (Figure 1). Thereafter, patients were disconnected from the mechanical ventilator and tracheal suction was performed with a 12F suction catheter-EMBRAMED™. After removing the suction catheter, patients were connected to the mechanical ventilator without any changing in the initial parameters.

**Figure 2:**
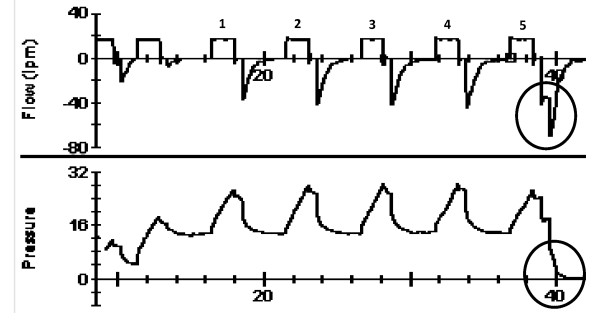
**Flow and pressure curves recorded during the PEEP-ZEEP technique, in circles, the moment of depressurization associated with manual bilateral thoracic compression**.

The values of PIP, Pplateau, Vinsp, and Vexp were registered during the five respiratory cycles. Manual bilateral thoracic compression was performed and registered at the 5th respiratory cycle for evaluate the PEEP-ZEEP technique's mechanism of action. All the measures were stored in a microcomputer equipped with Analysis Plus™ software (Figure 2). 

This protocol was approved by an Ethical Committee and all patients provided informed consent during the preoperative period.

### Statistical analysis

Descriptive analyses are presented as mean and standard deviation. When the variables along the time were normally distributed, One-way analysis of variance for repeated measures (ANOVA ONE-WAY RM) was used. Friedman Repeated Measures Analysis of Variance was used when variables were out of normality. Post-hoc Turkey test was also used, considering the significance level as p < 0.05.

Data were analyzed using the Statistical Package for Social Sciences™ for Windows™, 11.5 (SPSS Inc, Chicago, IL). Statistical significance was defined as p < 0.05.

## Results

**Figure 3:**
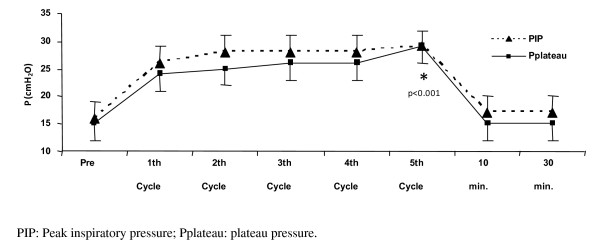
**Pressures before, at the 5 cycles, 10 and 30 minutes after the procedure**.

**Figure 4:**
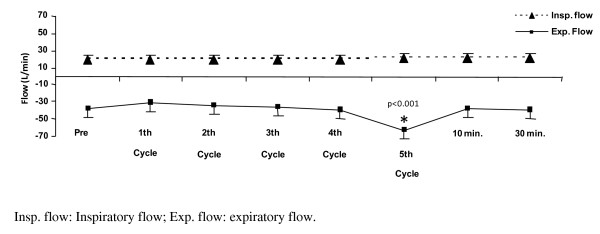
**Flows before, at the 5 cycles, 10 and 30 minutes after the procedure**.

Hemodynamic variables and S_p_O_2 _did not vary significantly before, 10 and 30 minutes after the PEEP-ZEEP technique (Table 2). The PIP was not different before, 10 minutes and 30 minutes after PEEP-ZEEP technique (p = 0.116) either. The PIP on the fifth respiratory cycle was 29 ± 3 cmH_2_O, and it was different between the previous values (p < 0.001) (Figure 3). The Pplateau also did not vary significantly before, 10 and 30 minutes after the PEEP-ZEEP technique. The Pplateau on the fifth respiratory cycle was 26 ± 3 cmH_2_O, greater than the other values (p < 0.001) (Figure 4).

**Table 2:** Mean values and standard deviation of mean before, 10 and 30 minutes after PEEP-ZEEP technique.

	Before	after 10 min	after 30 min	*p*
MAP (mmHg)	80 ± 12	82 ± 12	85 ± 18	0.749
HR (bpm)	97 ± 18	96 ± 16	97 ± 20	0.205
SpO_2 _(%)	97 ± 1	98 ± 2	98 ± 1	0.552
ETCO_2 _(mmHg)	37 ± 8	39 ± 9	40 ± 11	0.080
Rawinsp (cmH_2_O.s.L^-1^)	8 ± 2	8 ± 4	7 ± 3	0.197
Rawexp (cmH_2_O.s.L^-1^)	8 ± 2	8 ± 2	8 ± 2	0.977
Cst (mL/cmH_2_O)	46 ± 11	46 ± 13	44 ± 12	0.396

MAP: Mean arterial pressure; HR: heart rate; S_pO2_: peripheral oxygen saturation; ETCO_2_: end-tidal carbon dioxide; Rawinsp: inspiratory resistance; Rawexp: expiratory resistance; Cst: total static lung compliance. Values are presented as mean and standard deviation.

The inspiratory flow did not show difference before, at the fifth respiratory cycle, 10 minutes and 30 minutes after PEEP-ZEEP technique (p = 0.314).

The expiratory flow showed difference before the procedure (37 ± 11 L/min), at 10 minutes (38 ± 10 L/min) and at 30 minutes (39 ± 10 L/min) (p = 0.043). At the time of deflation, the expiratory flow reached 64 ± 9 L/min, with a significant statistical difference (p < 0.001) (Figures 3 and 4).

The inspiratory and expiratory resistance and static compliance parameters indicated slight oscillations, however with no significant statistical difference (table 2).

## Discussion

The main find of this study was that the PEEP-ZEEP technique was safe and did not cause significant alterations in hemodynamic variables, represented by HR and MAP. This safety may be related to the short period of pressurization (PEEP), as well as the given myocardial protection, immediately after the surgery, by the administration of inotropic and vasoactive drugs.

Initially, PEEP used was 5 cmH_2_O, which created a mean peak inspiratory pressure of 16 cmH_2_O. As it was increased 10 cmH_2_O to the initial PEEP (a total of 15 cmH_2_O), we maintained our patients' pressures within the safety limit recommended by a consensus/guideline of Mechanical Ventilation [7], even during the PEEP-ZEEP technique when a maximum peak pressure of 29 cmH_2_O was observed. This same pattern of change was observed on Pplateau.

As the airway pressurization was performed with PEEP at 15 cmH_2_O during 5 respiratory cycles because there was an expectation of a better gas redistribution and alveolar stabilization. Former studies suggest that PEEP values lower than 15 cmH_2_O, applied with similar characteristics as this protocol, were not enough to reopen collapsed areas [8,9]. With the airway pressurization at 15 cmH_2_O, an increase in functional residual capacity occurs, leading to a reduction in airway resistance and possibly helping on secretion removal [10,11].

During the study protocol, SpO_2 _and ETCO_2 _remained unaltered and it could be explained due to the fact that these patients had no previous pulmonary disease. Moreover our data are in accordance with the study by Kinloch [12] and Ackerman [13] who showed that the saline solution instillation did not alter PaO_2 _values.

In PEEP-ZEEP technique performed in this study, the significant increase in expiratory flow at the moment that PEEP was declined to zero was very evident. The sudden airway depressurization, associated with manual bilateral thoracic compression, as a part of PEEP-ZEEP technique, generated an increase in the exhaled tidal volume in a short expiratory time. This expiratory flow and exhaled tidal volume increase are in agreement with the hypothesis that the PEEP-ZEEP technique simulates the coughing mechanism.

It seems that one of the advantages of PEEP-ZEEP technique is the ability to simulate the cough, preventing early ventilator disconnection as seen in other techniques as bag squeezing. In bag squeezing technique, the peak inspiratory pressure depends on the size of the resuscitator in use, the operator's ability and differences equipment features that may vary in a significant form [14,15], what can expose patients to exponential risks. A positive benefit that PEEP-ZEEP technique has over other techniques is the peak inspiratory pressure control, providing safety to the patient.

In this study, values for inspiratory/expiratory resistance and static compliance were in agreement with the acceptable limits for intubated and mechanically ventilated patients, what could be influenced by the profile of the studied patients who did no show previous pulmonary alterations and underwent only few hours of mechanical ventilation.

The protocol was initiated 30 minutes after the patients were brought to the ICU as their ventilatory support lasted for less than 6 hours. These considerations, associated with the normal airway resistance, can justify the small amount of bronchial secretion removed with PEEP-ZEEP technique in this study. It is necessary to emphasize that the amount of bronchial secretion removed was not the aim of this study.

Our data show that the standardized PEEP-ZEEP technique did not produce significant alterations in hemodynamic and oxygenation parameters. However, it is known that pressurization at 15 cmH_2_O for a period over 15 minutes can alter the heart rate in patients submitted to cardiac surgery [11]. The differential in the present study was the short period of pressurization, approximately 25 seconds, what could have influenced the lack on hemodynamic alterations.

### Study limitation

This study is limited by the number of patients and by the fact that they did not show a great amount of secretion on respiratory system. Despite this, the amount of removed secretion was not the main endpoint of this study. A study comparing different techniques of secretion removal is required to understand the real benefits of PEEP-ZEEP technique in relation to other techniques.

## Conclusion

The PEEP-ZEEP technique seems to be safe, without alterations on hemodynamic variables, produces elevated expiratory flow and seems to be an alternative technique for the removal of bronchial secretions in patients submitted to a CABG.

## Competing interests

The authors declare that they have no competing interests.

## Authors' contributions

MVHR, MIZF, VOC and JOCAJ participated in the design and coordination of the study, MVHR participated in the data collection, MVHR, MIZF and VOC revised the manuscript, MVHR and VOC performed the statistical analysis. VOC, MVHR and MIZF participated in the writing. All authors read and approved the final version of the manuscript.
